# High-throughput living cell-based optical biosensor for detection of bacterial lipopolysaccharide (LPS) using a red fluorescent protein reporter system

**DOI:** 10.1038/srep36987

**Published:** 2016-11-14

**Authors:** Hui Jiang, Donglei Jiang, Jingdong Shao, Xiulan Sun, Jiasheng Wang

**Affiliations:** 1 State Key Laboratory of Food Science and Technology, School of Food Science and Technology, Synergetic Innovation Center of Food Safety and Nutrition, Jiangnan University, Wuxi, Jiangsu 214122, PR China; 2 School of Food Science and Technology, Jiangsu Key Labortary of Zoonoses, Yangzhou University, Yangzhou, Jiangsu 225127, PR China; 3 Zhangjiagang Entry-Exit Inspection And Quarantine Bureau, Zhangjiagang, Jiangsu 215600, PR China; 4 Univ Georgia, Dept Environm Hlth Sci, Athens, GA 30602, USA

## Abstract

Due to the high toxicity of bacterial lipopolysaccharide (LPS), resulting in sepsis and septic shock, two major causes of death worldwide, significant effort is directed toward the development of specific trace-level LPS detection systems. Here, we report sensitive, user-friendly, high-throughput LPS detection in a 96-well microplate using a transcriptional biosensor system, based on 293/hTLR4A-MD2-CD14 cells that are transformed by a red fluorescent protein (mCherry) gene under the transcriptional control of an NF-κB response element. The recognition of LPS activates the biosensor cell, TLR4, and the co-receptor-induced NF-κB signaling pathway, which results in the expression of mCherry fluorescent protein. The novel cell-based biosensor detects LPS with specificity at low concentration. The cell-based biosensor was evaluated by testing LPS isolated from 14 bacteria. Of the tested bacteria, 13 isolated Enterobacteraceous LPSs with hexa-acylated structures were found to increase red fluorescence and one penta-acylated LPS from Pseudomonadaceae appeared less potent. The proposed biosensor has potential for use in the LPS detection in foodstuff and biological products, as well as bacteria identification, assisting the control of foodborne diseases.

Gram-negative bacteria are the most common pathogens, and severely affect food/environmental safety and threaten public health. LPS, also called endotoxin, is an optimal biomarker for the detection of bacterial contamination, since it is the characteristic component of the outer membrane of Gram-negative bacteria. LPS induces extensive immune responses that can lead to fatal septic shock syndrome^1^,^2^,^3^,^4^, and can threaten human health even after bacterial death. Therefore, monitoring for the biological activity of endotoxin is as important as identification of the bacteria itself. Due to the high toxicity of LPS, significant effort has been directed toward the development of specific LPS detection systems to detect extremely small amounts of LPS for use in the medical, pharmacological and food security fields.

LPS measurement in biological samples is usually performed using gold standards approved and validated by the International Pharmacopoeia’s (FDA, 1987) Limulus Amebocyte Lysate (LAL) test. The LAL is an aqueous extract of blood cells (amoebocytes) from the horseshoe crab, *Limulus polyphemus*. The LAL extract reacts with LPS, and has a reported detection limit of around 10^−9^ g·mL^−1 ^5,6. This is an extremely sensitive limit that is useful for quantitative analysis, but due to the limitation of horseshoe crab (*Limulus polyphemus*) resources, and results of samples that tested negative using the LAL test but that were associated with adverse events determined by the use of Mono-Mac 6 cells and TLR expressing cells^7^, scientists are focusing their research on the development of complementary sensing methods for LPS detection. The sensitivity and selectivity of an assay are determined by molecules with specific affinity for LPS. These methods depend on selection of a recognition element, especially a bio-receptor or ligand. Several systems have been reported for the detection of LPS that use anti-LPS antibodies^8^ or antibiotics such as Polymyxin B^9^,^10^,^11^, and molecules designed specifically for LPS neutralization, namely aptamers^12^,^13^,^14^, LPS binding proteins^15^,^16^,^17^,^18^,^19^ or peptides^20^,^21^, and other synthetic LPS recognizing molecules^22^,^23^,^24^,^25^ as the recognition element. In addition to using natural and synthetic molecules with LPS affinity, living cells to detect LPS have been designed^7^,^26^.

Using whole living cells as the recognition element, cell-based biosensors provide a new strategy for *in vitro* tests^27^,^28^. External stimuli or changes in cellular microenvironment can perturb the “normal” physiological activities of mammalian cells, thus allowing cell-based biosensors to screen, monitor, and measure analyte-induced changes. Since the early works by Rechnitz and coworkers during the 1980 s^29^,^30^, numerous examples of combinations of a variety of cell species with various transducer devices or systems have been demonstrated. Giaever et al. reported an electrical mammalian cell biosensor that can continuously track morphological changes of adherent cells providing quantitative data from both sparse and confluent cultures^31^. Rider et al. reported the first cell sensor that used B lymphocytes to recognize specific bacteria with the help of membrane-bound IgM antibodies^32^. Mammalian cell-based biosensors have a distinct advantage in reflecting cellular physiological action rather than just quantitative detection and therefore can provide insight into mechanism of action of analytes^33^,^34^,^35^. More importantly, tests based on human cells rather than other cell types, are more likely to detect contaminants that can cause adverse effects in humans. Though prior work has examined the gross effects (e.g., viability, proliferation) of analytes on cells^31^,^36^,^37^, these gross assays do not reveal more subtle effects that mask or alter particular phenotypes of interest, such as the activation of signaling pathways. Recently, methods based on the reporting of analytes by fluorescence reaction in engineered cells offer a simple and nondestructive option^33^,^34^,^35^,^38^. Living cells used as biosensors are typically produced with a constructed plasmid, in which genes that code for the bio-reporter are placed under control of a promoter that recognizes the analyte of interest. Inducers activate the promoter genes, providing a genetic signal transducer that triggers and regulates the intensity of the bio-reporter’s expression.

LPS consists of three distinct regions: O-specific antigen, core polysaccharide and lipid A^39^. Of these, lipid A segment is the real pathogen-associated molecular pattern (PAMP). Lipid A is also a potent bacterial effector, and promotes activation of the innate immune system after binding the CD14 complex, myeloid differentiation protein 2 (MD-2) and toll-like receptor 4 (TLR4)^40^,^41^,^42^. LPS functions as an immuno-modulatory molecule that stimulates a strong innate immune response via the TLR4-MD2-CD14 pathway, resulting in the activation of nuclear factor κB (NF-κB), leading to up-regulation of co-stimulatory molecules and inflammatory cytokines^43^. There is a strong correlation between chemical structure of LPS, especially the lipid A structure, and the immunological response via the TLR4 pathway^44^,^45^. These functions are crucial components for the development of cell-based biosensors that use molecules with LPS affinity as an alternative for sensitive and accurate detection of LPS and evaluation of its inflammatory activity.

In developing a cell-based sensor, one must select the specificity of the sensor, or the set of stresses to which it will be responsive. In the present study, we describe the development and validation of a transcriptional biosensor system, based on 293/hTLR4A-MD2-CD14 cells transformed by a red fluorescent protein (mCherry) gene under the transcriptional control of an NF-κB response element. For the reporter, an mCherry fluorescent protein was selected that has a quick maturation time, good brightness, lack of oligomerization, and resistance to photo-bleaching^46^. The biosensor cells express mCherry fluorescence, allowing visual and nondestructive assessment of gene expression at single-cell resolution, while using commonly available equipment to quantify cellular fluorescence response without requiring a large numbers of cells. We describe the quantitative characterization of the biosensor, as well as its application to detect LPS extracted from different kinds of Gram-negative bacteria. The biosensor system presented here offers new possibilities for sensitive and effective assessment of bacterial LPS presence in a high-throughput, non-invasive manner, with no need for sophisticated equipment.

## Results

### Working pinciples of the cell-based biosensor

**Figure 1:**
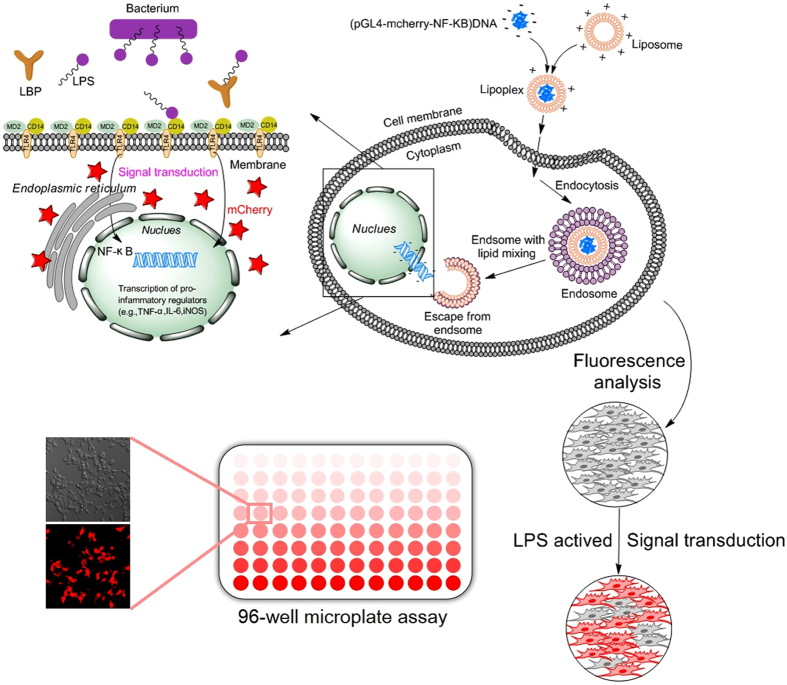
Schematic illustration of the working principle of the cell-based biosensor.

The biosensor cells are 293/hTLR4A-MD2-CD14pGL4.26-mcherry-NF-κB, transfected with multiple plasmids that express the human endotoxin receptor, TLR4, co-receptors (myeloid differentiation protein-2, MD2 and cluster of differentiation-14, CD14) and the mCherry reporter protein under the control of transcription factor NF-κB, a principal transcription regulator for a variety of gene-encoding cytokines, chemokines, adhesion, and co-stimulatory molecules. Figure 1 illustrates the working principle. During lipid-mediated, DNA-transfection of the 293/hTLR4A-MD2-CD14 cell sensor, DNA-lipid complexes fuse with the cell membrane by endocytosis and the fluorescent protein gene diffuses through the intracellular membranes to enter the nucleus. When the biosensor cells are exposed to LPS, TLR4 and co-receptors recognize the LPS and trigger the signaling cascade that leads to activation of NF-κB and subsequent expression of mCherry fluorescent protein (see the enlarged drawing on the left of Fig. 1). Using this assay, we monitored cell responses and quantified LPS levels by monitoring intracellular fluorescence changes as observed under an ImageXpress^®^ Micro XLS Widefield High Content Screening (HCS) (Molecular Devices, USA). The assay is optimized for a 96-well microplate format to allow HCS quantification of induced mCherry expression.

### Successful construction of the cell-based biosensor

The plasmid, pGL4.26-mCherry-NF-κB, was constructed with the NF-κB response element inserted in front of the mCherry reporter gene. Target gene fragments were successfully synthesized (see Supplementary Fig. S1), and inserted into the pGL4.26-mCherry plasmid, thus generating the novel recombinant plasmid, pGL4.26-mCherry-NF-κB (see Supplementary Fig. S2A). By applying restriction analysis, successful construction and isolation of the pGL4.26-mCherry-NF-κB plasmid was confirmed. One-cut digestion with *BlpI* confirmed the length of this recombinant plasmid to be 5034 bp (see Supplementary Fig. S2B).

Next, the pGL4.26-mcherry-NF-κB plasmid was transfected to 293/hTLR4A-MD2-CD14 cells. Western blot was used to detect mCherry protein expression by 293/hTLR4A-MD2-CD14 cells that were exposed to LPS standard, both with and without transfection by pGL4.26-mCherry-NF-κB carrying anti-mCherry antibodies. One major band was observed at ~28.8 kDa (see Supplementary Fig. S3A). Compared to non-transfected cells, the 293/hTLR4A-MD2-CD14 cells transfected with pGL4.26-mCherry-NF-κB showed strong fluorescence signals from mCherry expression after LPS activation (see Supplementary Fig. S3B). Using fluorescence-activated cell sorter (FACS), results were also validated for spectrofluorimetric quantification of the induction of mCherry expression in these 293/hTLR4A-MD2-CD14pGL4.26-mcherry-NF-κB cells. Compared to non-transfected cells, the fluorescence intensity histogram of transfected cells after LPS activation exhibits a change in the peak to higher fluorescence intensity (see Supplementary Fig. S3C). These results together suggest successful construction of a cell-based LPS detection biosensor with a red fluorescent protein reporter.

To investigate potential adverse effects during transfection, MTT-based proliferation assays were performed. As shown in Supplementary Fig. S4A, results showed no difference in cellular vitality between the transfected and non-transfected groups (t-test, *p* > 0.05) at 1, 2, 3, 4 and 5 days. By staining cells with annexin V-FITC and PI, FACS was used to distinguish and to quantitatively determine the percentages of dead, viable, apoptotic and necrotic cells after transfection. As shown in Supplementary Fig. S4B, results of FACS analysis of apoptosis in non-transfected and transfected cells indicated similar low rates of early apoptosis (0.05% versus 0.12%, respectively) and necrosis (0.18% versus 0.21%, respectively).

### Verification of the dependence of mCherry red fluorescent protein expression on TLR4-pathway activation

**Figure 2:**
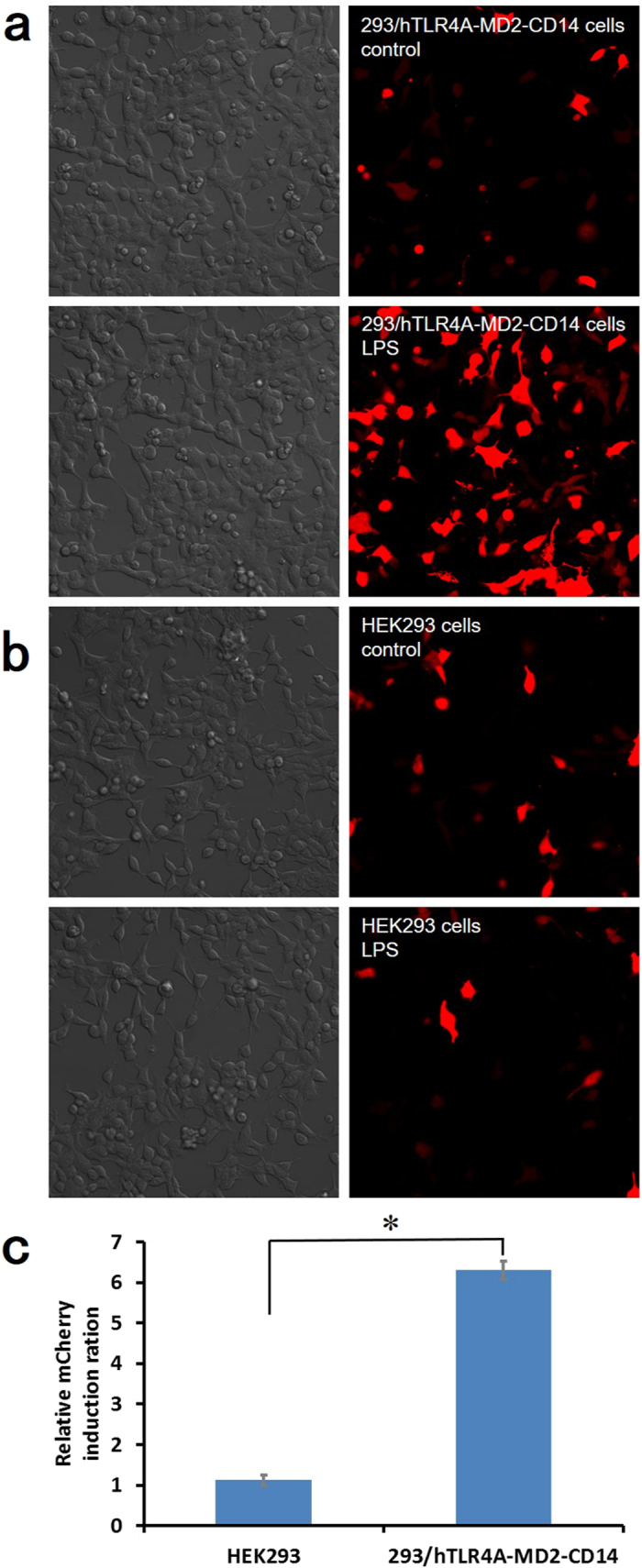
(**a**) Bright and fluorescence images of 293/hTLR4A-MD2-CD14 cells transfected pGL4.26-mCherry-NF-κB without (control) and with (LPS) exposure to 100 ng·mL^−1^ LPS. 293/hTLR4A-MD2-CD14 cells exhibit red fluorescence. (**b**) Bright and fluorescence images of HEK293 cells transfected pGL4.26-mCherry-NF-κB without (control) and with (LPS) exposure to 100 ng·mL^−1^ LPS for 24 h. HEK293 cells rarely exhibit red fluorescence. (**c**) Relative induction ratio of red fluorescence of pGL4.26-mCherry-NF-κB transfected cells in response to LPS. Data are represented relative to untreated control cells as mean ± s.e.m. and *p*-value obtained by independent-samples t-test. The degree of significance is indicated when appropriate (*p < 0.05, n = 3).

As shown in Fig. 2a, a clear increase in mCherry red fluorescence was observed for LPS when compared to the control (medium without LPS), which confirms the sensing principle. To further assess the specificity of our biosensor, we examined its response to HEK293 cells, both with and without the TLR4-pathway. To do this, HEK293 cells, both with and without the TLR4-pathway, were transfected with pGL4.26-mCherry-NF-κB. Next, HEK293 cells that stably expressed the TLR4/MD-2/CD14 cassette (293/TLR4/MD-2/CD14), or TLR4-negative HEK293 cells, were treated with medium or stimulated with LPS for 24 h. 293/hTLR4A-MD2-CD14 cells transfected with pGL4.26-mCherry-NF-κB turned red upon LPS induction, whereas no red fluorescence was observed in transfected HEK293 cells (Fig. 2a,b). As shown in Fig. 2c, relative mCherry induction ratio was 6.30 ± 0.23 in 293/hTLR4A-MD2-CD14 cells and was significantly reduced to 1.12 ± 0.12 in HEK293 cells (t-test, *p* = 0.0080, n = 3), indicating inhibition of mCherry expression in cells without the TLR4-pathway. This inhibition indicates that the TLR4-pathway is required for red fluorescent protein expression in our biosensors, consistent with the NF-κB-dependent expression of mCherry red fluorescent protein.

As HEK293 cells express endogenous levels of TLR3, TLR5 and NOD1, 293/hTLR4A-MD2-CD14pGL4.26-mcherry-NF-κB cells will respond to their cognate ligands, such as poly(I:C), flagellin and C12-iE-DAP, respectively (see Supplementary Fig. S5). Therefore, in the blind detection, in order to identify TLR4-specific responses, we recommend to use 293/pGL4.26-mcherry-NF-κB cells as a control cell line. LPS in samples with TNF or interleukins may interfere with the detection of this system. Therefore, this system does not apply to blood samples.

### Quantitative characterization of the cell-based biosensor using LPS standard

**Figure 3:**
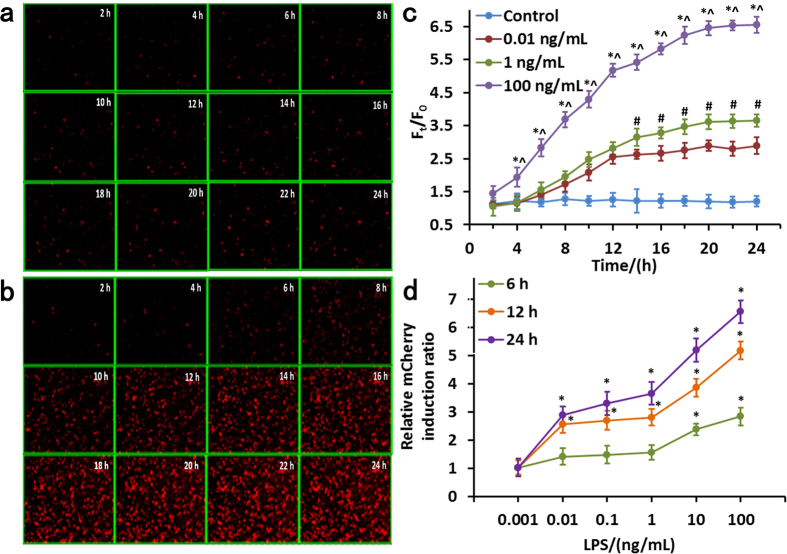
Time- and dose-dependent induction of mCherry expression in biosensor cells treated with LPS standard. Time-lapse images of cell responses without (**a**) and with (**b**) exposure to 1 ng·mL^−1^ LPS. (**c**) Time-dependence curves of mCherry expression in biosensor cells in response to LPS. All data shown is mean ± s.e.m. and *p*-value obtained by One-way ANOVA (*p < 0.05, n = 3). (**d**) Relative induction ratio of mCherry expression in biosensor cells treated with increasing LPS concentrations; analyzed using HCS after 6, 12, 24 h exposure. All data shown is mean ± s.e.m. and *p*-value obtained by independent-samples t-test (*p < 0.05, n = 3).

We next characterized the time- and dose-dependence of the cell-based biosensor response to LPS standard. To determine the amplitude and speed of cell responses, fluorescence intensity changes by HCS in real-time were monitored and recorded using time-lapse imaging of cell responses before (Fig. 3a) and after (Fig. 3b) exposure to LPS (1 ng·mL^−1^). Before exposure to LPS, there was no change in fluorescence (Fig. 3a), but as shown in Fig. 3b, red fluorescence increased with time of exposure to LPS. This observed increase of mCherry red fluorescence in response to LPS was time-dependent, as reflected in rising F_t_/F_0_ values over time. In cells exposed to 0.01 ng·mL^−1^ LPS, the measured F_t_/F_0_ value increased from1.42-fold after 6 h, to 2.56-fold after 12 h of exposure, and then to 2.90-fold after 24 h of exposure. In cells exposed to 1 ng·mL^−1^ LPS, the measured F_t_/F_0_ value increased from 1.55-fold after 6 h, to 2.81-fold after 12 h of exposure, and to 3.66-fold after 24 h of exposure. In cells exposed to 100 ng·mL^−1^ LPS, the measured F_t_/F_0_ value increased from 2.84-fold after 6 h, to 5.18-fold after 12 h of exposure, and to 6.55-fold after 24 h of exposure. The resulting curves indicate that the F_t_/F_0_ increased rapidly during early incubation and it grew slowly later, reaching the highest response at an LPS exposure time of 20 h. At longer exposures, cells displayed no additional increased induction of red fluorescence, instead reaching a plateau. The induced expression level of reporter genes was relatively stable over the tested exposure periods. The control (medium without LPS) cells did not induce mCherry red fluorescent protein expression in any tested period (Fig. 3c). Change in biosensor cells with 100 ng·mL^−1^ LPS significantly increased their F_t_/F_0_ at >4 h, in contrast to biosensor cells with 1 ng·mL^−1^ and 0.01 ng·mL^−1^ LPS, respectively (One-way ANOVA, *,^*p* < 0.05, n = 3). By contrast, there were no significant differences in F_t_/F_0_ values between biosensor cells with 1 ng·mL^−1^ LPS and 0.01 ng·mL^−1^ LPS before 14 h (One-way ANOVA, *p* > 0.05, n = 3), which result demonstrates the difficulty of discerning at short stimulation time between responses to different doses or concentrations at low concentration. At exposure times of ≥14 h, it was possible to detect the induction level of red fluorescence at lower concentrations. Therefore, most applications will obtain optimal read-out at 20 h following exposure.

Next, we examined the dose-dependence of the cell-based biosensor response to LPS. On the basis of the experimental results stated above, we selected three time nodes (6 h, 12 h and 24 h) for further research. Figure 3d shows the curves at different LPS induction times. The responses depend on the LPS concentration and the induction time for LPS. There is nearly no red fluorescence produced for treatment with 0.001 ng·mL^−1^ LPS after any exposure time. LPS concentrations above 10 ng·mL^−1^ caused significant red fluorescence induction compared to the controls after 6 h exposure (t-test, *p < 0.05, n = 3). Higher LPS concentrations caused a dose-dependent increase of induced mCherry red fluorescent protein levels. LPS induced a significant dose-dependent increase in mCherry fluorescence after 12/24 h and with ≥0.01 ng·mL^−1^ LPS (t-test, *p < 0.05, n = 3). In addition, relative mCherry induction rate increased gradually at low concentrations (<1 ng·mL^−1^) and grew quickly at high concentrations (>1 ng·mL^−1^).

**Figure 4:**
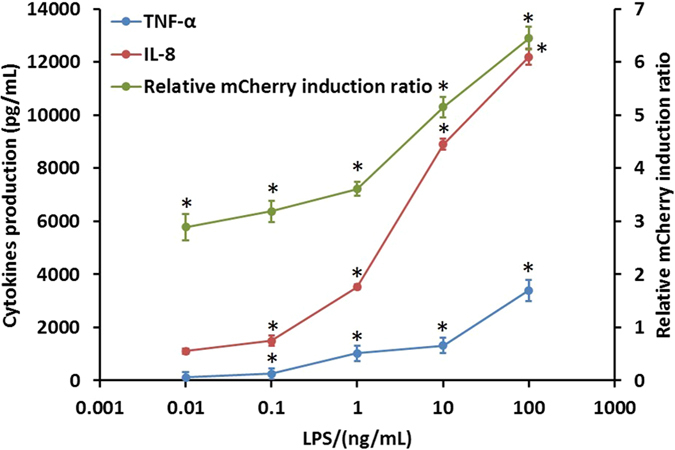
Relative mCherry induction ratio and the changes of cytokine production (TNF-α and IL-8) after 20 h exposure of biosensor cells to LPS standard. All data shown is mean ± s.e.m. and *p*-value obtained by independent-samples t-test (*p < 0.05, n = 3).

To further evaluate the suitability of NF-κB induction as an identifying element for LPS-induced TLR4-pathway activation, we determined the parallel, LPS-induced increase of mCherry red fluorescent protein expression and cytokine production after challenging the cells with LPS at 20 h exposure. In the experiment, a range of concentrations of LPS standard was used to induce NF-κB-dependent mCherry expression, with a lowest concentration of 0.01 ng·mL^−1^. The relationship between the logarithmic value of LPS concentration and the relative mCherry fluorescence induction ratio at 20 h exposure clearly demonstrates that fluorescence signals follow the same trend as LPS-activated cells at other given points in time. The relative mCherry fluorescence induction ratio was proportional to LPS, ranging from 0.01 to 100 ng·mL^−1^. LPS induced significant rise in the relative mCherry fluorescence induction ratio after 20 h exposure at all concentrations (t-test, *p < 0.05, n = 3). At the highest tested LPS concentration (100 ng·mL^−1^), there was a 6.45-fold increase in the relative mCherry fluorescence induction ratio over the control (Fig. 4). As shown in Fig. 4, the biosensor cells began to secrete TNF-α and IL-8 at an LPS concentration of 0.01 ng·mL^−1^. LPS did not induce a significant increase in TNF-α and IL-8 production at 0.01 ng·mL^−1^ (t-test, *p* > 0.05, n = 3), but at LPS concentrations above 0.1 ng·mL^−1^, the production of TNF-α and IL-8 significantly increased dose-dependently (t-test, *p < 0.05, n = 3). The parallel measurement of TNF-α and IL-8 production during exposure to LPS shows the same trend as that of relative mCherry fluorescence induction ratio. Overall, the data indicate that NF-κB is a suitable identification element for LPS that induces TLR4-pathway activation.

### Response of Biosensors exposure to LPS from several bacterial species

**Figure 5:**
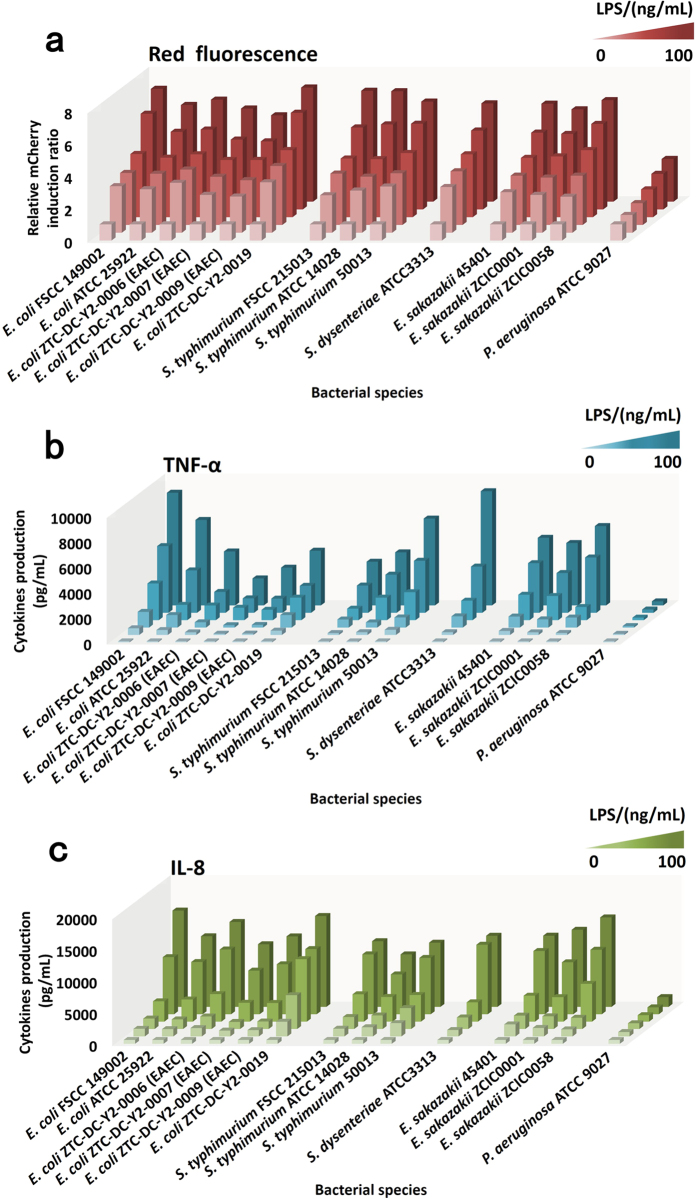
Relative mCherry induction ratio (**a**), cytokine TNF-α production (**b**), and cytokine IL-8 production (**c**) after 20 h exposure of biosensor cells to LPS from several bacterial species. The concentrations of LPS from low to high in order are: 0, 0.01, 0.1, 1, 10 and 100 ng·mL^−1^.

Previous studies confirmed that LPS standard from *E. coli* 055:B5 induced a dose-dependent increase of red fluorescence, as indicated by the increased values of relative mCherry induction ratio (Fig. 3d). To study the response of the constructed biosensors, we extracted LPS from two standard strains of *E. coli* and four wild strains of *E. coli* from food samples. Similarly, after 20 h exposure, the challenge to the biosensor cells with LPS significantly up-regulated the expression of the mCherry red fluorescent protein in treated cells compared to untreated cells under increased LPS concentration (Fig. 5, see Supplementary Table S1). This increase in fluorescence correlated with increased TNF-α and IL-8 production.

Recently, it was found that the acylation state of lipid A determines whether or not LPS is recognized by the human TLR4-MD2 complex^47^,^48^,^49^. To further assess the sensitivity of this cell-based biosensor for detection of LPS, we tested several kinds of extracted LPS with known lipid A structures.

All Gram-negative bacteria express LPS in their outer membranes, but the exact LPS structures vary. To investigate whether LPS isolated from bacterial species other than *E. coli* induce mCherry red fluorescent protein expression in biosensor cells, we stimulated biosensor cells with LPS from *S. typhimurium, S. dysenteriae, E. sakazakii* (Enterobacteriaceae), and *P. aeruginosa* (Pseudomonadaceae). SDS-PAGE analysis revealed that all LPSs from several selected bacterial species have an intact O-antigen (see Supplementary Fig. S6), therefore interference by polysaccharides with receptor recognition of the lipid A moiety can be ignored^50^.

Hexa-acylated LPS that were isolated from the enterobacterial species induced mCherry red fluorescent protein expression, TNF-α, and IL-8 production to a level comparable with that of treatment with LPS from *E. coli* (Fig. 5). After 20 h exposure, in the range of 0.01 to 100 ng·mL^−1^, all tested LPS isolated from the enterobacterial species exhibited significantly increased values for relative mCherry induction ratio (t-test, *p < 0.05, n = 3), and production of TNF-α and IL-8 improved accordingly (Fig. 5, see Supplementary Table S1). In contrast, penta-acylated LPS isolated from *P. aeruginosa* was obviously less potent, inducing only weak mCherry red fluorescent protein expression and very small increases in TNF-α and IL-8. LPS from *P. aeruginosa* was unable to significantly induce mCherry red fluorescent protein expression (t-test, *p* > 0.05, n = 3, Fig. 5, see Supplementary Table S1), or affect TNF-α and IL-8 production below 1 ng·mL^−1^. Even at 100 ng·mL^−1^, activation of biosensor cells with *P. aeruginosa* LPS only resulted in a 2.64-fold relative mCherry induction ratio. These experiments demonstrated that the penta-acylated LPS structure of *P. aeruginosa* displayed a low ability to engage the TLR4-mediated signaling.

## Discussion

Conceived, tested and validated in this study is a proposed transcriptional, live cell-based optical biosensor system for detecting bacterial LPS with better combined visual confirmation, nondestructive execution, high-throughput and user-friendly features. This transcriptional biosensor reports the TLR4-mediated cellular response to LPS stimulation via NF-κB-driven mCherry protein expression. We engineered biosensor cells to use mCherry red fluorescence protein due to its quick maturation time, good brightness, lack of oligomerization, and resistance to photo-bleaching, endowing the novel biosensor with stability in cells and good spectral characteristics, with excitation/emission in the red region of the spectrum, preventing overlap with cell autofluorescence. We chose 293/hTLR4A-MD2-CD14 cells as the background because they are very easy to culture and transfect, plus they express the TLR4-MD2-CD14 protein and have been extensively studied for the biological activity of LPS^39^,^51^,^52^, showing sensitivity to different sources of LPS (structure variation).

In validating the dependence of mCherry red fluorescence protein expression on TLR4-pathway activation, we determined the ratio of the fluorescence of biosensor cells without TLR4 and co-receptors with LPS to the ratio produced by biosensor cells with TLR4 and co-receptors after exposure to LPS. We validated that expression of mCherry red fluorescence protein expression depends on TLR4-pathway activation, and then we characterized our biosensor using LPS standard.

To determine the dynamics of time and dose responses, the biosensor cells were monitored up to 24 h and exposed to a range of LPS concentrations, from very low concentrations that induced little fluorescence response to higher concentrations. The results indicated that a higher response signal could be obtained by extending LPS stimulation time. This suggested that additional mCherry red fluorescent protein expression by cells increased the ratio to increase biosensor sensitivity; the response signal stabilized after 20 h. The use of LPS standard at sufficiently high concentration induced an obvious response of the biosensor after exposure over any time period. However, a short stimulation time at low concentrations resulted in only weak differences of F_t_/F_0_ values between different doses. Experimental results show that the optimal exposure time for detection of mCherry expression is 20 h. The tests using varying LPS concentration showed a dose-dependent increase of induced mCherry red fluorescent protein levels, except for 0.001 ng·mL^−1^ LPS. Relative mCherry induction ratio showed a rapid and significant increase at LPS concentrations above 1 ng·mL^−1^ compared to the controls at any exposure time (t-test, *p < 0.05, n = 3).

To confirm the fluorescence analysis results, a cytokine ELISA assay was performed. Figure 4 indicates that TNF-α and IL-8 production rates increased gradually at low LPS concentrations and increased rapidly at high concentrations. These data agree well with the dose-dependent curve as obtained from fluorescence measurements. Hence, NF-κB is suitable as an identification element for LPS that induces TLR4-pathway activation, as confirmed by previous research^43^.

To illustrate uses for different sources of LPS, we exposed biosensor cells to LPS that was isolated from Enterobacteriaceae and Pseudomonadaceae. Slight changes in LPS lead to specific structural diversity of LPS^50^ and we evaluated the effect of these structural difference on the response by the biosensors. To do this, we selected LPS from *E. coli, S. typhimurium, S. dysenteriae*, and *E. sakazakii* (Enterobacteriaceae), and *P. aeruginosa* (Pseudomonadaceae). Our results show that after 20 h exposure, TLR4 response on the surface of the biosensor cells was specific to LPS, allowing forced response to the LPSs isolated from enterobacterial species, such as *E. coli, S. typhimurium, S. dysenteriae*. The statistical analysis of *p*-values shows significant differences between tested LPS isolated from enterobacterial species and the control for this biosensor (t-test, *p* < 0.05, n = 3; see Supplementary Table S1). In view of the above-mentioned facts, these results are likely due to the conserved structures for lipid A among the Enterobacterial species. It has been reported that LPS, as isolated from most bacteria in the family Enterobacteriaceae, such as *E. coli, S. typhimurium, S. dysenteriae*, and *E. sakazakii*, contains six acyl-chains^50^. Changes in the degree of lipid A acylation have a dramatic effect on the binding of this molecule to MD-2 and TLR4. The hexa-acylated lipid A species exhibits optimal inflammatory activity^39^, the lipid A with five lipid chains is 100-fold less active, and versions of lipid A with four lipid chains completely lack agonistic activity^53^,^54^. As expected, the treatment with penta-acylated LPS from *P. aeruginosa* did not show significant increase in the fluorescence ratio when compared to the control for this biosensor at levels below 1 ng·mL^−1^ (t-test, *p* > 0.05, n = 3). This lack of induced pGL4.26-mcherry-NF-κB reporting of LPS by *P. aeruginosa* is consistent with a recent study demonstrating that LPS isolated from *P. aeruginosa* is a poor agonist for human TLR4, due to its penta-acylated structure^49^.

The main advantages of this biosensor system are: (i) the use of the TLR4 pathway in cells allows extremely sensitive, direct and nondestructive detection and recognition of inflammatory activity of LPSs from different Gram-negative bacteria; (ii) the use of mCherry autofluorescence allows a low cost and easily performed cell-based biosensor assay without any chromogenic reagent; and (iii) the use of HCS measurement of fluorescence in microplates allows visualization, automatic operation, real-time monitoring and high-throughput data acquisition. The main hurdle that remains is that the cells themselves are not comparably ‘stable’ against other biosensing elements, such as antibodies or nucleic acid probes. The signal response varies from batch to batch, and is influenced mainly by the cell’s viability and the exposing time. To overcome this hurdle, the early-passage numbers (passage numbers before 10) of cells are preferred, and controls must be used for each test batch.

The validation of the test performed here demonstrates that this biosensor system is sensitive at ng·mL^−1^ levels, can selectively distinguish inflammatory activity in response to LPS, based on the expression of mCherry fluorescent protein, and can be used for basic initial prediction of differences between LPS molecular structures (mainly the number of acyl chains in lipid A). For comparison purposes, we summarized recent reports using different recognition elements and approaches for LPS sensing in Table 1. Compared with other techniques, the proposed method has impressive results and the detection limit is lower than some other works. This method that uses the NF-κB response element and the mCherry reporter gene suggests promising future development of practical, cell-based biosensors for various applications including LPS detection in foodstuff and biological products and the identification of bacteria in food safety and medicine.

## Methods

### Cell line and cell culture

293/hTLR4A-MD2-CD14 cells, obtained from InvivoGen, were cultured in Dulbecco’s Modified Eagle’s Medium (DMEM) supplemented with 4.5 g·L^−1^ glucose, 10% (v/v) fetal bovine calf serum, 50 U·ml^−1^ penicillin, 50 μg·ml^−1^ streptomycin, 100 μg·ml^−1^ Normocin^TM^, 2 mM L-glutamine. Cells were grown at 37 °C in a humidified incubator with 5% CO_2_. In this study, the early-passage numbers (passage numbers before 10) of 293/hTLR4A-MD2-CD14 cells were used for the cell-based biosensor development.

### Engineering the Cell-Based Biosensor

A reporter gene construct encoding the mCherry fluorescent protein, the expression of which is under the transcriptional control of a nuclear factor kappa B (NF-κB) response element, was engineered using standard molecular biology techniques of restriction and ligation. The eukaryotic transcription factor NF-κB was identified as a protein that bound to a specific decameric DNA sequence GGG ACT TTC C^55^.

To create the pGL4.26-mCherry-NF-κB reporter construct, the following oligonucleotides were synthesized: oligonucleotide 1,5′-TCAGGGACTTTCTTCAAATCCGGGACTTTC-3′ and oligonucleotide 2,5′-TGAGAAAGTCCCGGATTTGAAGAAAGTCCC-3′. In addition, the pGL4.26 [luc2/minP/Hygro] plasmid (Promega) was used for sub-cloning, to provide the appropriate restriction sites on each side of the *mCherry* sequence. This was achieved by replacing the *luc2* coding sequence in pGL4.26 with *mCherry*, to create the pGL4.26-mCherry plasmid. Finally, the oligonucleotides were phosphorylated, annealed, and ligated into the *BlpI* site of the recombinant vector pGL4.26-mCherry which generated a new plasmid, pGL4.26-mCherry-NF-κB. Sequencing was performed by sending the new plasmid to Sangon Biotech (Shanghai, China). The newly constructed pGL4.26-mCherry-NF-κB plasmid was cloned into the DH5α *E. coli* strain and isolated using SanPrep Column Enodotoxin-Free Plasmid Mini-Preps Kit (Sangon Biotech, Shanghai, China), according to manufacturer’s instructions.

The purified plasmids were subjected to quality control using agarose gel electrophoresis. The recombinant plasmids were digested with *BlpI* restriction enzyme (New England Biolabs Inc).

The day before transfection, cells were trypsinized and counted. Approximately 1×10^6^ cells were plated per well of 6-well plate (Corning Costar, New York, USA) in 2 mL of complete growth medium. Transfection of cells was carried out at 70–90% of cell confluency. To this end, 3.75 μL of Lipofectamine^®^ 3000 (Invitrogen) was diluted into 250 μL Opti-MEM (Gibco). 2.5 μg of pGL4.26-mCherry-NF-κB DNA was diluted into 250 μL Opti-MEM, then 5 μL P3000^TM^ was added. After that diluted DNA was added to the above diluted Lipofectamine^®^ 3000 (1:1 ratio). 250 μL of the DNA-lipid complexes were incubated for 5 min then were added to each well containing cells and complete growth medium (medium without penicillin and streptomycin) according to the manufacturer’s instructions. Then, 6 h post-transfection, the medium was replaced with 2 ml of new complete growth medium. Assessment of the red florescence protein for tracking the 293/hTLR4A-MD2-CD14 cells expressing mCherry was performed using an excitation wavelength/bandwidth of 590/20 nm and an emission wavelength/bandwidth of 645/40 nm with an HCS, through a 20 × Plan Fluor objective.

### LPS preparation and SDS-PAGE analysis

Eight standard strains (*E. coli* ATCC 25922, *E. coli* FSCC 149002, *S. typhimurium* 50013, *S. typhimurium* FSCC 215013, *S. typhimurium* ATCC 14028, *P. aeruginosa* ATCC 9027, *E. sakazakii* 45401 and *S. dysenteriae* ATCC3313) and six strains from food samples were obtained from Zhangjiagang Entry-Exit Inspection and Quarantine Bureau (Suzhou, China). All of the strains were grown in Luria-Bertani broth medium (usb, Cleveland, USA) at 37 *°*C in shaker incubator overnight. After centrifugation of culture media, sedimented bacteria were harvested and used for LPS extraction and purification.

LPS was obtained from the aqueous phase of a water-phenol extract according to a published procedure^56^. To remove traces of nucleic acids or proteins that could interfere with endotoxin detection, LPS extracts were dialyzed, lyophilized and purified following published protocols^57^.

For SDS-PAGE analysis, LPS was suspended in sterile ddH_2_O to achieve an appropriate concentration. Ten microliters of each concentrated supernatant preparation was run on 4% stacker and 12.5% vertical resolving gels against different kinds of LPS. Gels were run in a Tris-Tricine running buffer (Bio-Rad, Hercules, Calif.). LPS were visualized by using a previously described silver staining protocol^58^.

### Response of the cell-based biosensors exposure to LPS

The TLR4 signaling pathway was activated by TLR ligand-tested LPS standard: LPS from *E. coli* 055:B5 (protein <1%, RNA <1%, catalogue number L4524, Sigma Corp., St. Louis, MO, USA). According to the solubility of the LPS standard, the stock solution was dissolved in cell culture medium without fetal bovine calf serum supplement at a concentration of 1 mg·mL^−1^. Immediately before use, further dilutions were performed in complete growth medium.

The assays were performed in black, clear-bottomed, 96-well microtitre plates (Corning Costar 3603, New York, USA). Aliquots of 293/hTLR4A-MD2-CD14pGL4.26-mCherry-NF-κB cell suspensions (100 μL; 5 × 10^5^ cells·mL^−1^) were distributed into all 96 wells of the microtitre plates. To allow attaching, the biosensor cells were incubated for 4 h, then washed with PBS, prior to the LPS exposure.,The test LPS at the appropriate concentrations was added to each well, then incubated in the plates at 37 °C in 5% CO_2_. The control cells were exposed to the same medium without LPS and to the same number of washes. The highest tested concentration of LPS standard was set at 100 ng·mL^−1^. Cellular images of response to the LPS-activated TLR4 signaling pathway were assessed using mCherry fluorescence by HCS with a 10 × Plan Fluor objective. At different time points and doses after exposure, the red fluorescence response of the biosensor cells was determined. Time-lapse sequences of biosensor cells, recorded every 2 h, were collected, and the total fluorescence intensity for each image was analyzed using ImageJ (NIH, US) software. In time-dependent experiments, we designated F_t_ as the measure of mCherry fluorescence at a given time point, and F_0_ as the stable baseline of mCherry fluorescence before LPS addition. The change in mCherry fluorescence over time was quantified by the curve of F_t_/F_0_ against time. In dose-dependent experiments, response is presented as relative mCherry induction ratio, which is the ratio between mCherry fluorescence of the treated cells and background fluorescence by non-treated control cells. Background mCherry fluorescence change was analyzed using a blank sample of DMEM and regarded as noise level.

### Determination of TNF-α and IL-8

Supernatants were collected and stored at −70 °C until TNF-α and IL-8 determination. Levels of TNF-α and IL-8 were assayed using a standard Enzyme Linked Immunosorbent Assay (ELISA) kit (NeoBioscience Technology Co., Ltd. Beijing, China). The assays were carried out exactly according to the supplier’s instructions.

### Statistical analysis

All values are presented as mean ± s.e.m. One-way ANOVA (LSD) or independent-samples t-test was used for statistical analysis using software (SPSS Statistics 21.0 for Windows, IBM Corp., NY, USA). A value of *p* < 0.05 was considered statistically significant.

## Additional Information

**How to cite this article**: Jiang, H. *et al*. High-throughput living cell-based optical biosensor for detection of bacterial lipopolysaccharide (LPS) using a red fluorescent protein reporter system. *Sci. Rep.* **6**, 36987; doi: 10.1038/srep36987 (2016).

**Publisher’s note**: Springer Nature remains neutral with regard to jurisdictional claims in published maps and institutional affiliations.

## Supplementary Material

Supplementary Information

## Figures and Tables

**Table 1:** Comparison of several reported methods for LPS detection.

Recognition element	Detection technology	Sensitivity	Ref.
LAL	Magnetoelastic sensor	0.0105 EU·mL^−1^	59
Endothelial cells	EIS	0.5 μg·mL^−1^	26
Antibody	Chemiluminescence	0.01–10 ng·mL^−1^	8
N,N-Dimethyl-N-(pyrenyl-1-methyl) dodecan-1-ammonium	Fluorescent	100 nmol·L^−1^	23
Recombinant factor C zymogen	DPV	1000–5000 EU·L^−1^	16
Aptamer	EIS	1 pg·mL^−1^	12
Polymyxin B	CV	1 μg·mL^−1^	9
Poly-ε-lysine	CV	2 ng·mL^−1^	15
T4 phage adhesin	Microwave	100 pg·mL^−1^	18
293/hTLR4A-MD2-CD14pGL4.26-mcherry-NF-κB cells.	Fluorescent	0.01 ng·mL^−1^	This method
