# Supplementary material for: High-throughput living cell-based optical biosensor for detection of bacterial lipopolysaccharide (LPS) using a red fluorescent protein reporter system
Source: Sci Rep. 2016 Nov 14;6:36987. doi: 10.1038/srep36987 (PMC5107890; doi:10.1038/srep36987)
Supplement: Supplementary Information [file srep36987-s1.pdf]

Supplementary information:

# High-throughput living cell-based optical biosensor for detection of bacterial lipopolysaccharide (LPS) using a red fluorescent protein reporter system

**Hui Jiang [1], Donglei Jiang [2], Jingdong Shao [3], Xiulan Sun [1, *], Jiasheng Wang [1, 4, *]**

*[1] State Key Laboratory of Food Science and Technology, School of Food Science and Technology, Synergetic Innovation Center of Food Safety and Nutrition, Jiangnan University, Wuxi, Jiangsu 214122, PR China*

*[2] School of Food Science and Technology, Jiangsu Key Labortary of Zoonoses, Yangzhou University, Yangzhou, Jiangsu 225127, PR China*

*[3] Zhangjiagang Entry-Exit Inspection And Quarantine Bureau, Zhangjiagang, Jiangsu 215600, PR China*

*[4] Univ Georgia, Dept Environm Hlth Sci, Athens, GA 30602 USA*

*Correspondence and requests for materials should be addressed to X.S. (email: sxlzzz@jiangnan.edu.cn).*

*1.1 Western blot*

Proteins from the cells were harvested with Western lysis buffer containing PMSF. The protein concentration was determined by Bicinchoninic Acid (BCA) assay. Equal amounts of proteins were separated using 12% SDS-PAGE after being boiled for 5 min in 4 × loading buffer and transferred onto nitro cellulose (NC) membrane. The transferred membranes were incubated with anti-mCherry antibody (abcam, ab125096). Then second antibody IgG-HRP (abcam, ab6789). Immunoreactivity was detected using the ECL detection system (GE Healthcare Bio-Sciences Corp. NJ).

*1.2 Determination of cell viability*

MTT assay was performed to determine cell viability. 293/hTLR4A-MD2-CD14 cells and 293/hTLR4A-MD2-CD14pGL4.26-mCherry-NF-κB cells were seeded in 96-well plates (Corning Costar, New York, USA) at an initial density of 5000 cells well$^{-1}$ for several hours. At 4 h before the end of incubation, 10 μl of MTT (5 mg mL$^{-1}$ in PBS) (Beyotime Institute of Biotechnology, Nantong, Jiangsu, China) was added to each well, and incubated for 4 h. After the medium was removed at the end of incubation, 150 μl of DMSO (dimethylsulfoxide) was added to each well, and shaken at room temperature for 10 min until the absorbance was measured at 490 nm using an Synergy 2 Multi-Mode Microplate Reader (BioTek, Winooski, VT, USA). The results were presented as the average values of three runs.

*1.3 Annexin V-FITC/PI stained fluorescence-activated cell sorter (FACS)*

At 3 days after transfection, transfected and non-transfected cells were harvested through trypsinization, and washed twice with cold PBS (0.15 mol L$^{-1}$, pH 7.2). The cells were centrifuged at 800 r/min for 5 min, then the supernatant was discarded and the pellet was resuspended in 1 × binding buffer at a density of 1.0 × 10$^{5}$-1.0 × 10$^{6}$ cells mL$^{-1}$. 100 μL of the sample solution was transferred to a 5 mL culture tube,

and incubated with 5 μL of FITC-conjugated annexin V and 5 μL of PI for 15 min at room temperature in the dark. Four hundred μL of 1 × binding buffer was added to each sample tube, and the samples were analyzed by BD Biasciences FACS Calibur Flow Cytometry (BD Biasciences, NJ, USA) using Cell Quest Research Software.

**Figures Caption:**

**Fig.S1** Sequencing results.

**Fig.S2** Schematic representation of the pGL4.26-mCherry-NF-κB plasmid construction (A), and confirmation of the successful construction of the plasmid using restriction analysis (B). Lane 1, restriction carried out with the restriction enzyme *BlpI* ; Lane 2, DNA ladder Marker.

**Fig.S3** Expression of the mCherry protein in 293/hTLR4A-MD2-CD14 cells exposed to LPS standard after transfection and non-transfection with the pGL4.26-mCherry-NF-κB plasmid. (A) Western blotting of lysates prepared from cells with anti-mCherry antibodies: M, protein marker; lane1, negative control (non-transfected cells); lane 2, mCherry protein (transfected cells). (B) Fluorescence microscopy of cells showing mCherry signal (left: rare fluorescence, right: red fluorescence). (C) FACS analysis of fluorescence intensity histograms of cells.

**Fig.S4** Cell viability after transfection. (A) The growth curve of cellular vitality of non-transfected cells and transfected cells determined by MTT assay. All data shown is mean ± s.e.m. and *p*-value obtained by independent-samples t-test (*$p < 0.05$, n = 3). (B) FACS analysis of apoptosis in non-transfected (left) and transfected cells (right). The lower left quadrant contains annexin V-FITC (-) and PI (-) viable cells; the lower right quadrant, annexin V-FITC (+) and PI (-) early apoptotic cells; the upper right quadrant, annexin V-FITC (+) and PI (+) late apoptotic or necrotic cells; the upper left quadrant, annexin V- FITC (-) and PI (+) necrotic cells.

**Fig.S5** Overlay images (bright and fluorescence) of 293/hTLR4A-MD2-CD14 cells transfected pGL4.26-mCherry-NF-κB with exposure to poly (I:C), flagellin and C12-iE-DAP, respectively. 293/hTLR4A-MD2-CD14 cells exhibit red fluorescence.

**Fig.S6** SDS-PAGE of LPS. 1: *E. coli* ZTC-DC-Y2-0006 (EAEC); 2: *E. coli*

ZTC-DC-Y2-0007 (EAEC); 3: *S. typhimurium* 50013; 4: *E. coli* 055:B5 (LPS standard); 5: *E. coli* ATCC 25922; 6: *P. aeruginosa* ATCC 9027; 7: *E. coli* FSCC 149002; 8: *E. coli* ZTC-DC-Y2-0009 (EAEC); 9: *E. sakazakii* 45401; 10: *E. coli* ZTC-DC-Y2-0019; 11: *S. typhimurium* FSCC 215013; 12: *S. typhimurium* ATCC 14028; 13: *E. sakazakii* ZCIC0058; 14: *E. sakazakii* ZCIC0001; 15: *S. dysenteriae* ATCC3313.

**Fig.S7** Relative mCherry induction ratio of the biosensor cells incubated using different concentrations of LPS standard (0.01, 0.1, or 1 ng $mL^{-1}$) for 20 h.

**Table S1** Relative mCherry induction ratio and cytokine production (TNF-α and IL-8) after 20 h exposure of biosensor cells to LPS from several bacterial species.

**Table S2** Precision (indicated by relative standard deviation, RSD) of LPS standard (20 h) detection by the proposed sensor (n=5).

**Table S3** Reproducibility (indicated by RSD) of LPS standard (1.0 ng $mL^{-1}$, 20 h) detection by the proposed sensor.

GTGCAAGCAGTGCAGACTTTCTCTGGCCTAACTGGCCGGTACCTGAGCTCGCTAGCCTCGAGGATATCAAGATCTGGCCTCGGCGGCCAAGCT

TGCTCAGGGACTTTCTTCAAATCCGGGACTTTCTCAGGGACTTTCTTCAAATCCGGGACTTTCTCAGGGACTTTCTTCAAATCCGGGACTTTC

TCAGCAGACACTAGAGGGTATATAATGGAAGCTCGACTTCCAGCTTGGCAATCCGGTACTGTTGGTAAAGCCACCATGGTGAGCAAGGGCGAG

GAGGATAACATGGCCATCATCAAGGAGTTCATGCGCTTCAAGGTGCACATGGAGGGCTCCGTGAACGGCCACGAGTTCGAGATCGAGGGCGAG

GGCGAGGGCCGCCCCTACGAGGGCACCCAGACCGCCAAGCTGAAGGTGACCAAGGGTGGCCCCCTGCCCTTCGCCTGGGACATCCTGTCCCCT

CAGTTCATGTACGGCTCCAAGGCCTACGTGAAGCACCCCGCCGACATCCCCGACTACTTGAAGCTGTCCTTCCCCGAGGGCTTCAAGTGGGAG

CGCGTGATGAACTTCGAGGACGGCGGCGTGGTGACCGTGACCCAGGACTCCTCCCTGCAGGACGGCGAGTTCATCTACAAGGTGAAGCTGCGC

GGCACCAACTTCCCCTCCGACGGCCCCGTAATGCAGAAGAAGACCATGGGCTGGGAGGCCTCCTCCGAGCGGATGTACCCCGAGGACGGCGCC

CTGAAGGGCGAGATCAAGCAGAGGCTGAAGCTGAAGGACGGCGGCCACTACGACGCTGAGGTCAAGACCACCTACAAGGCCAAGAAGCCCGTG

CAGCTGCCCGGCGCCTACAACGTCAACATCAAGTTGGACATCACCTCCCACAACGAGGACTACACCATCGTGGAACAGTACGAACGCGCCGAG

GGCCGCCACTCCACCGGCGGCATGGACGAGCTGTACAAGTAATTCTAGAGTCGGGGCGGCCGGCCGCTTCGAGCAGACATGATAGATACATTG

ATGAGTTTGGACAACCACACTAGGATGCAGTGAAAAAATGCTTTATTTGTGAAATTGTGATGCTATTGCTTTATTTTAACCATTAAAAGCTGC

AATAAACAAGTTAAAAACCAAAATTGCATCATTAAG

**Fig.S1** Sequencing results.

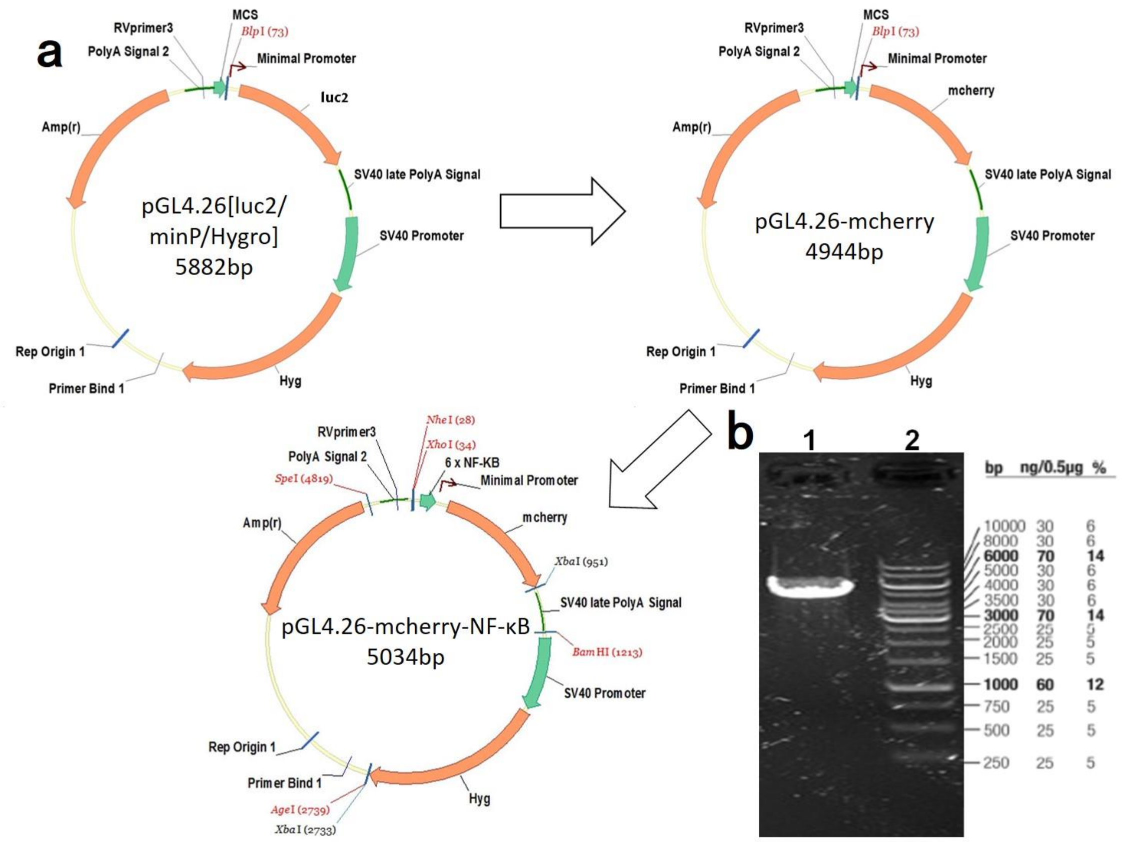


**Fig.S2** Schematic representation of the pGL4.26-mCherry-NF-κB plasmid construction (A), and confirmation of the successful construction of the plasmid using restriction analysis (B). Lane 1, restriction carried out with the restriction enzyme *BlpI* ; Lane 2, DNA ladder Marker.

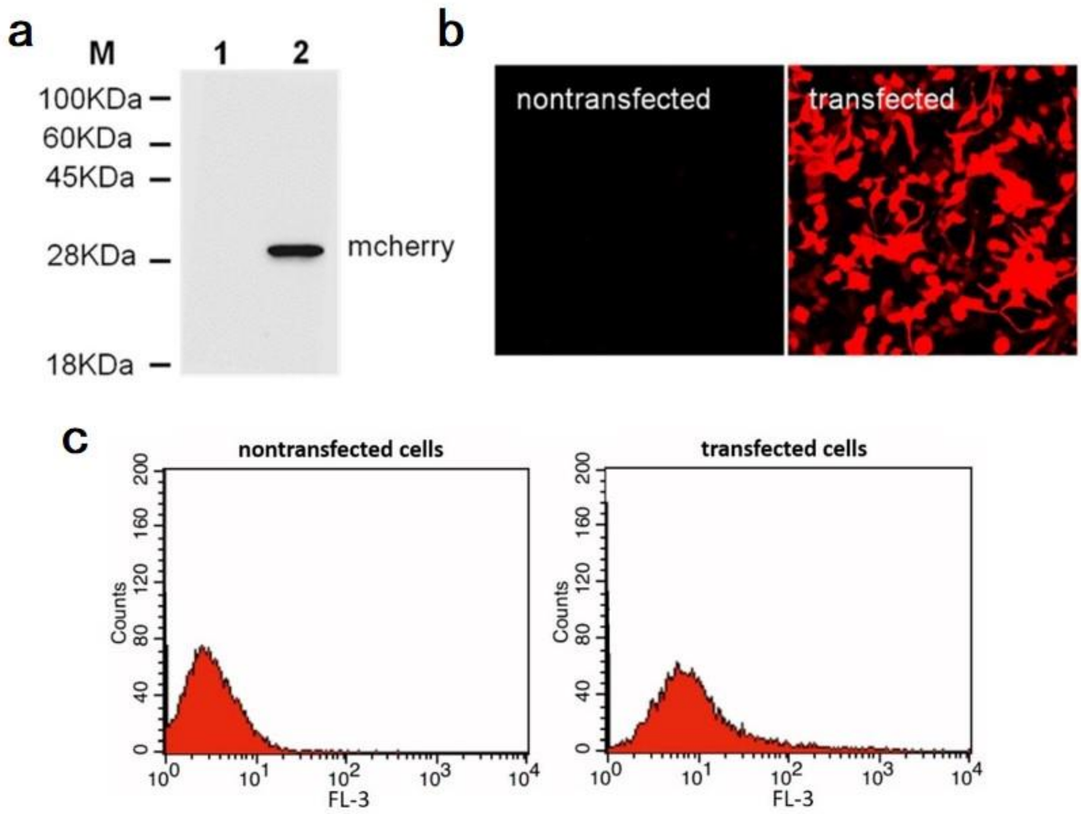


**Fig.S3** Expression of the mCherry protein in 293/hTLR4A-MD2-CD14 cells exposed to LPS standard after transfection and non-transfection with the pGL4.26-mCherry-NF-κB plasmid. (A) Western blotting of lysates prepared from cells with anti-mCherry antibodies: M, protein marker; lane1, negative control (non-transfected cells); lane 2, mCherry protein (transfected cells). (B) Fluorescence microscopy of cells showing mCherry signal (left: rare fluorescence, right: red fluorescence). (C) FACS analysis of fluorescence intensity histograms of cells.

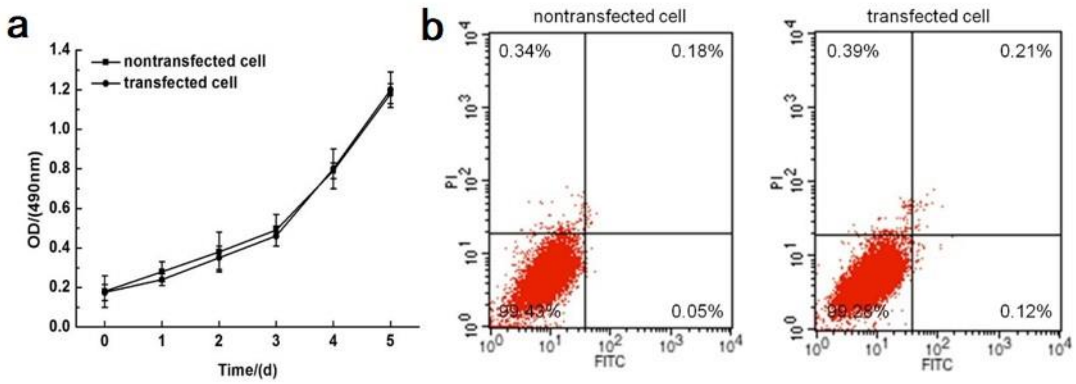


**Fig.S4** Cell viability after transfection. (A) The growth curve of cellular vitality of non-transfected cells and transfected cells determined by MTT assay. All data shown is mean ± s.e.m. and *p*-value obtained by independent-samples t-test ($*p < 0.05$, n = 3). (B) FACS analysis of apoptosis in non-transfected (left) and transfected cells (right). The lower left quadrant contains annexin V-FITC (-) and PI (-) viable cells; the lower right quadrant, annexin V-FITC (+) and PI (-) early apoptotic cells; the upper right quadrant, annexin V-FITC (+) and PI (+) late apoptotic or necrotic cells; the upper left quadrant, annexin V- FITC (-) and PI (+) necrotic cells.

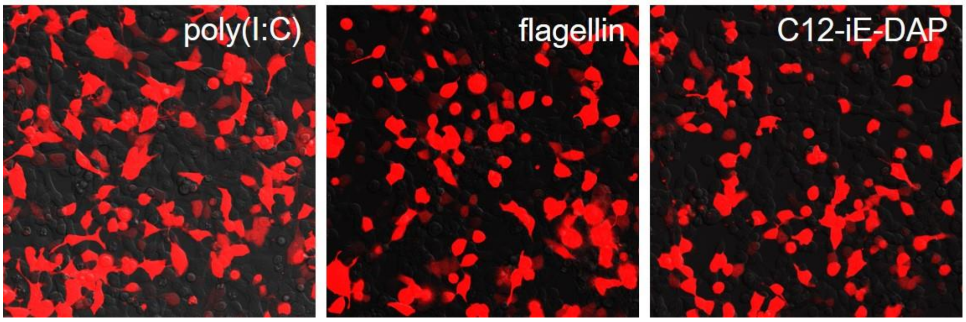


**Fig.S5** Overlay images (bright and fluorescence) of 293/hTLR4A-MD2-CD14 cells transfected pGL4.26-mCherry-NF-κB with exposure to poly (I:C), flagellin and C12-iE-DAP, respectively. 293/hTLR4A-MD2-CD14 cells exhibit red fluorescence.

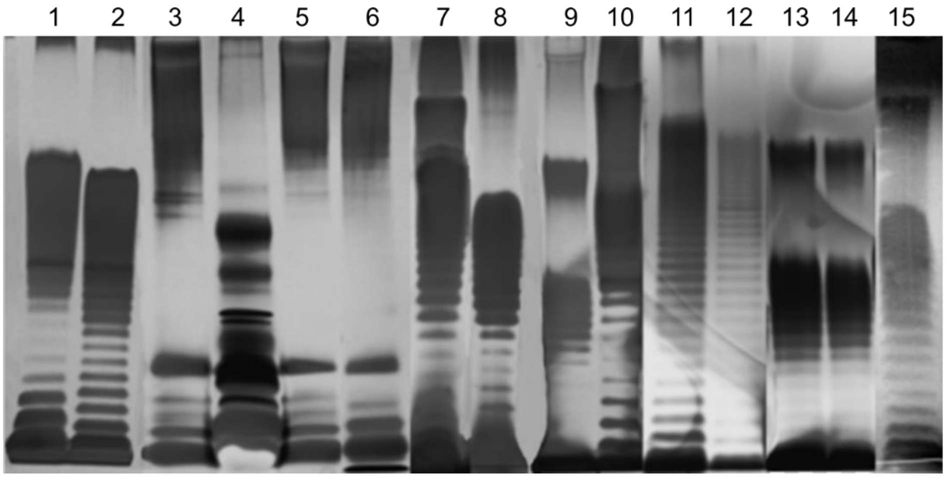


**Fig.S6** SDS-PAGE of LPS. 1: *E. coli* ZTC-DC-Y2-0006 (EAEC); 2: *E. coli* ZTC-DC-Y2-0007 (EAEC); 3: *S. typhimurium* 50013; 4: *E. coli* 055:B5 (LPS standard); 5: *E. coli* ATCC 25922; 6: *P. aeruginosa* ATCC 9027; 7: *E. coli* FSCC 149002; 8: *E. coli* ZTC-DC-Y2-0009 (EAEC); 9: *E. sakazakii* 45401; 10: *E. coli* ZTC-DC-Y2-0019; 11: *S. typhimurium* FSCC 215013; 12: *S. typhimurium* ATCC 14028; 13: *E. sakazakii* ZCIC0058; 14: *E. sakazakii* ZCIC0001; 15: *S. dysenteriae* ATCC3313.

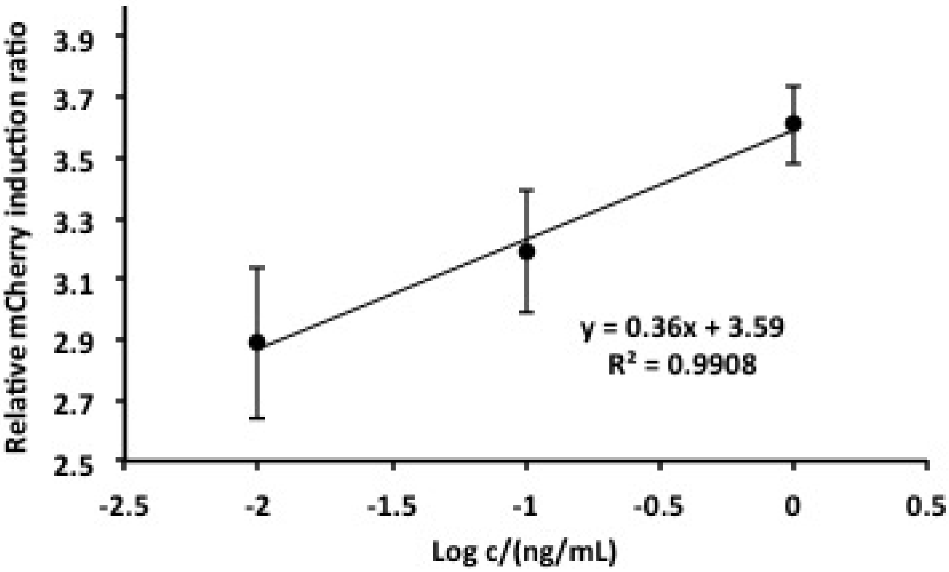


**Fig.S7** Relative mCherry induction ratio of the biosensor cells incubated using different concentrations of LPS standard (0.01, 0.1, or 1 ng mL$^{-1}$) for 20 h.

As shown in Supplementary Fig. S7 online, a linear behavior between the relative mCherry induction ratio and the log of the LPS standard concentration was obtained from 0.01 to 1 ng mL$^{-1}$ ($R^2$ = 0.9908).

**Table S1** Relative mCherry induction ratio and cytokine production (TNF-α and IL-8) after 20 h exposure of biosensor cells to LPS from several bacterial species.

| **Bacterial species** | **Number** | **Con. ng/mL[a]** | **mCherry ind. ±s.e.m.[b]** | **Cytokine production ±s.e.m. (ng/mL)[c]** | |
|---|---|---|---|---|---|
| | | | | TNF-α | IL-8 |
| ***E. coli*** | FSCC 149002 | 0 | 1.00 ±0.09 | 0.08 ±0.00 | 0.61 ±0.01 |
| | | 0.01 | 2.88 ±0.23 | 0.53±0.10 | 1.20 ±0.11 |
| | | 0.1 | 3.22 ±0.19 | 1.24 ±0.09 | 1.65 ±0.09 |
| | | 1 | 3.91 ±0.11 | 2.90 ±0.15 | 3.21 ±0.17 |
| | | 10 | 5.92 ±0.18 | 5.27 ±0.16 | 9.02 ±0.23 |
| | | 100 | 6.98 ±0.21 | 8.90 ±0.17 | 15.21 ±0.14 |
| | ATCC 25922 | 0 | 1.00 ±0.09 | 0.08 ±0.00 | 0.61 ±0.01 |
| | | 0.01 | 2.69 ±0.21 | 0.39 ±0.09 | 1.15 ±0.09 |
| | | 0.1 | 3.17 ±0.15 | 0.99 ±0.02 | 1.52 ±0.05 |
| | | 1 | 3.68 ±0.20 | 1.21 ±0.01 | 3.50 ±0.14 |
| | | 10 | 4.80 ±0.14 | 3.35 ±0.09 | 8.25 ±0.12 |
| | | 100 | 5.98 ±0.18 | 6.75 ±0.05 | 11.15 ±0.26 |
| | ZTC-DC-Y2-0006 (EAEC) | 0 | 1.00 ±0.09 | 0.08 ±0.00 | 0.61 ±0.01 |
| | | 0.01 | 3.09 ±0.23 | 0.21 ±0.05 | 1.32 ±0.10 |
| | | 0.1 | 3.43 ±0.17 | 0.42 ±0.07 | 1.98 ±0.19 |
| | | 1 | 3.89 ±0.21 | 1.16 ±0.09 | 4.35 ±0.08 |
| | | 10 | 4.94 ±0.13 | 1.65 ±0.11 | 10.23 ±0.13 |
| | | 100 | 6.31 ±0.21 | 4.26 ±0.18 | 13.41 ±0.27 |
| | ZTC-DC-Y2- | 0 | 1.00 ±0.09 | 0.08 ±0.00 | 0.61 ±0.01 |

| | | | | | |
|---|---|---|---|---|---|
| | 0007 (EAEC) | 0.01 | 2.34 ±0.24 | 0.10 ±0.01 | 0.92 ±0.09 |
| | | 0.1 | 2.98 ±0.14 | 0.19 ±0.07 | 1.12 ±0.11 |
| | | 1 | 3.54 ±0.25 | 0.98 ±0.09 | 2.98 ±0.19 |
| | | 10 | 4.32 ±0.15 | 1.14 ±0.05 | 6.89 ±0.21 |
| | | 100 | 5.76 ±0.11 | 2.13 ±0.12 | 9.89 ±0.22 |
| | ZTC-DC-Y2-0009 (EAEC) | 0 | 1.00 ±0.09 | 0.08 ±0.00 | 0.61 ±0.01 |
| | | 0.01 | 2.24 ±0.13 | 0.11 ±0.02 | 0.99 ±0.09 |
| | | 0.1 | 2.77 ±0.09 | 0.21 ±0.01 | 1.19 ±0.11 |
| | | 1 | 3.54 ±0.19 | 0.82 ±0.06 | 2.95 ±0.05 |
| | | 10 | 4.21 ±0.21 | 1.12 ±0.09 | 7.89 ±0.13 |
| | | 100 | 5.34 ±0.15 | 2.98 ±0.11 | 11.13 ±0.11 |
| | ZTC-DC-Y2-0019 | 0 | 1.00 ±0.09 | 0.08 ±0.00 | 0.61 ±0.01 |
| | | 0.01 | 3.12 ±0.14 | 0.34 ±0.03 | 2.32 ±0.13 |
| | | 0.1 | 3.65 ±0.25 | 0.99 ±0.05 | 5.34 ±0.12 |
| | | 1 | 4.15 ±0.11 | 1.78 ±0.11 | 9.87 ±0.22 |
| | | 10 | 5.98 ±0.19 | 2.13 ±0.10 | 10.29 ±0.29 |
| | | 100 | 7.06 ±0.18 | 4.32 ±0.21 | 14.35 ±0.21 |
| ***S. typhimurium*** | FSCC 215013 | 0 | 1.00 ±0.09 | 0.08 ±0.00 | 0.61 ±0.01 |
| | | 0.01 | 2.32 ±0.21 | 0.15 ±0.01 | 1.22 ±0.04 |
| | | 0.1 | 3.18 ±0.17 | 0.64 ±0.04 | 1.90 ±0.11 |
| | | 1 | 3.63 ±0.11 | 0.91 ±0.03 | 4.32 ±0.15 |
| | | 10 | 5.07 ±0.15 | 2.15 ±0.10 | 9.46±0.24 |
| | | 100 | 6.86 ±0.19 | 3.45 ±0.11 | 10.37 ±0.28 |
| | ATCC 14028 | 0 | 1.00 ±0.09 | 0.08 ±0.00 | 0.61 ±0.01 |

| | | | | | |
|---|---|---|---|---|---|
| | | 0.01 | 2.61 ±0.17 | 0.22 ±0.03 | 1.45 ±0.02 |
| | | 0.1 | 2.99 ±0.11 | 0.41 ±0.01 | 2.14 ±0.23 |
| | | 1 | 3.59 ±0.15 | 1.78 ±0.09 | 3.90 ±0.10 |
| | | 10 | 5.26 ±0.12 | 3.01 ±0.13 | 6.29 ±0.29 |
| | | 100 | 6.83 ±0.22 | 4.19 ±0.21 | 8.29 ±0.21 |
| | 50013 | 0 | 1.00 ±0.09 | 0.08 ±0.00 | 0.61 ±0.01 |
| | | 0.01 | 2.86 ±0.17 | 0.41 ±0.01 | 2.10 ±0.07 |
| | | 0.1 | 3.20 ±0.14 | 0.80 ±0.06 | 3.31 ±0.03 |
| | | 1 | 3.97 ±0.21 | 2.21 ±0.07 | 4.21 ±0.10 |
| | | 10 | 5.30 ±0.16 | 4.11 ±0.24 | 8.90 ±0.23 |
| | | 100 | 6.19 ±0.13 | 6.86 ±0.29 | 10.14 ±0.26 |
| ***S. dysenteriae*** | ATCC 3313 | 0 | 1.00 ±0.09 | 0.08 ±0.00 | 0.61 ±0.01 |
| | | 0.01 | 2.83 ±0.21 | 0.22 ±0.02 | 1.01 ±0.02 |
| | | 0.1 | 3.32 ±0.17 | 0.89 ±0.09 | 1.83 ±0.01 |
| | | 1 | 3.89 ±0.13 | 1.54 ±0.12 | 3.05 ±0.13 |
| | | 10 | 4.88 ±0.22 | 3.65 ±0.11 | 10.98 ±0.32 |
| | | 100 | 6.08 ±0.19 | 9.02 ±0.22 | 11.23 ±0.34 |
| ***E. sakazakii*** | 45401 | 0 | 1.00 ±0.09 | 0.08 ±0.00 | 0.61 ±0.01 |
| | | 0.01 | 2.51 ±0.13 | 0.32 ±0.04 | 1.90 ±0.05 |
| | | 0.1 | 3.05 ±0.22 | 0.87 ±0.05 | 2.12 ±0.09 |
| | | 1 | 3.67 ±0.25 | 2.01 ±0.17 | 4.10 ±0.14 |
| | | 10 | 4.75 ±0.19 | 3.92 ±0.15 | 9.99 ±0.21 |
| | | 100 | 6.06 ±0.14 | 5.34 ±0.13 | 11.24 ±0.29 |
| | ZCIC0001 | 0 | 1.00 ±0.09 | 0.08 ±0.00 | 0.61 ±0.01 |

| | | | | | |
|---|---|---|---|---|---|
| | | 0.01 | 2.34 ± 0.14 | 0.21 ± 0.02 | 1.34 ± 0.09 |
| | | 0.1 | 2.93 ± 0.25 | 0.65 ± 0.01 | 2.01 ± 0.05 |
| | | 1 | 3.76 ± 0.21 | 1.91 ± 0.21 | 3.87 ± 0.11 |
| | | 10 | 4.67 ± 0.15 | 3.14 ± 0.09 | 8.21 ± 0.21 |
| | | 100 | 5.71 ± 0.17 | 4.93 ± 0.25 | 12.18 ± 0.22 |
| | ZCIC0058 | 0 | 1.00 ± 0.09 | 0.08 ± 0.00 | 0.61 ± 0.01 |
| | | 0.01 | 2.23 ± 0.14 | 0.16 ± 0.04 | 1.11 ± 0.02 |
| | | 0.1 | 3.05 ± 0.17 | 0.79 ± 0.13 | 1.78 ± 0.06 |
| | | 1 | 4.15 ± 0.19 | 1.04 ± 0.07 | 5.98 ± 0.24 |
| | | 10 | 5.29 ± 0.09 | 4.37 ± 0.22 | 10.19 ± 0.32 |
| | | 100 | 6.28 ± 0.16 | 6.27 ± 0.21 | 14.13 ± 0.19 |
| ***P. aeruginosa*** | ATCC 9027 | 0 | 1.00 ± 0.09 | 0.08 ± 0.00 | 0.61 ± 0.01 |
| | | 0.01 | 1.10 ± 0.12 | 0.09 ± 0.03 | 0.69 ± 0.02 |
| | | 0.1 | 1.35 ± 0.17 | 0.12 ± 0.05 | 0.91 ± 0.03 |
| | | 1 | 1.72 ± 0.24 | 0.20 ± 0.06 | 1.02 ± 0.02 |
| | | 10 | 2.20 ± 0.11 | 0.26 ± 0.16 | 1.09 ± 0.01 |
| | | 100 | 2.64 ± 0.23 | 0.32 ± 0.19 | 1.54 ± 0.07 |

[a] Biosensor cells were treated with ten-fold dilution series of the LPS solution.

[b] Relative mCherry induction expressed as the ratio between the mCherry fluorescence intensity of the treated cells and non-treated control cells.

[c] Following an 20 h incubation at 37 ℃, supernatant was recovered and analyzed for respective cytokines by enzyme-linked immunosorbent assay (ELISA) according to the manufactures directions (NeoBioscience Technology Co., Ltd. Beijing, China).

Grey areas represent significantly different values compared to control ($p < 0.05$, n = 3).

All data shown is mean $\pm$ s.e.m. and *p*-value obtained by independent-samples t-test.

**Table S2** Precision (indicated by relative standard deviation, RSD) of LPS standard (20 h) detection by the proposed sensor (n=5).

| Spiked (ng mL$^{-1}$) | Found (ng mL$^{-1}$) | Recovery (%) | RSD (%) |
|---|---|---|---|
| 0.02 | 0.0197 | 98.5 | 2.12 |
| 0.1 | 0.0992 | 99.2 | 2.80 |
| 0.9 | 0.9158 | 101.8 | 2.45 |

The samples were directly spiked with 0.02 ng mL$^{-1}$, 0.1 ng mL$^{-1}$, and 0.9 ng mL$^{-1}$ LPS standard, respectively, and then the samples were analyzed by the developed method. The results obtained for the analyzed samples are summarized in Supplementary Table S2 online. The recoveries were acceptable for all the samples tested and %RSD was less than 2.80%. Therefore, the established method can be used to detect LPS.

**Table S3** Reproducibility (indicated by RSD) of LPS standard (1.0 ng mL$^{-1}$, 20 h) detection by the proposed sensor.

| | | Relative mCherry induction ratio | Average relative mCherry induction ratio | RSD (%) |
|---|---|---|---|---|
| Biosensor cells via three separate transfection | 1 | 3.68 | 3.61 | 2.51 |
| | 2 | 3.65 | | |
| | 3 | 3.51 | | |
| Biosensor cells via single transfection | 1 | 3.68 | 3.61 | 2.16 |
| | 2 | 3.66 | | |
| | 3 | 3.65 | | |
| | 4 | 3.60 | | |
| | 5 | 3.59 | | |
| | 6 | 3.63 | | |
| | 7 | 3.41 | | |
| | 8 | 3.66 | | |
| | 9 | 3.58 | | |
| | 10 | 3.65 | | |

To evaluate the reproducibility of the proposed sensor, experiments were carried out in 1.0 ng mL$^{-1}$ LPS standard solution at 20 h exposure. The cell-based biosensor was prepared with a relative mCherry induction ratio RSD of 2.51% using three separate transfected biosensor cells. The excellent reproducibility was obtained with a %RSD of 2.16% (calculated using sensors, from a single transfection process) after 10 measurements. The RSDs can be seen in Supplementary Table S3 online.
